# Knockdown of SALL4 inhibits the proliferation and reverses the resistance of MCF-7/ADR cells to doxorubicin hydrochloride

**DOI:** 10.1186/s12867-016-0055-y

**Published:** 2016-03-02

**Authors:** Yuan-Yuan Chen, Zhi-Zhen Li, Yuan-Yuan Ye, Feng Xu, Rui-Jie Niu, Hong-Chen Zhang, Yi-Jian Zhang, Ying-Bin Liu, Bao-San Han

**Affiliations:** Department of General Surgery and Laboratory of General Surgery, Xinhua Hospital, Affiliated with Shanghai Jiao Tong University, School of Medicine, No. 1665 Kong Jiang Road, 200092 Shanghai, China; Institute of Biliary Tract Disease, Shanghai Jiao Tong University, School of Medicine, 200092 Shanghai, China

**Keywords:** SALL4, Proliferation, Chemo-resistance, Breast cancer, Doxorubicin

## Abstract

**Background:**

Breast cancer is the most frequent malignancy in women and drug resistance is the major obstacle for its successful chemotherapy. In the present study, we analyzed the involvement of an oncofetal gene, sal-like 4 (SALL4), in the tumor proliferation and drug resistance of human breast cancer.

**Results:**

Our study showed that SALL4 was up-regulated in the drug resistant breast cancer cell line, MCF-7/ADR, compared to the other five cell lines. We established the lentiviral system expressing short hairpin RNA to knockdown SALL4 in MCF-7/ADR cells. Down-regulation of SALL4 inhibited the proliferation of MCF-7/ADR cells and induced the G1 phase arrest in cell cycle, accompanied by an obvious reduction of the expression of cyclinD1 and CDK4. Besides, down-regulating SALL4 can re-sensitize MCF-7/ADR to doxorubicin hydrochloride (ADMh) and had potent synergy with ADMh in MCF-7/ADR cells. Depletion of SALL4 led to a decrease in IC50 for ADMh and an inhibitory effect on the ability to form colonies in MCF-7/ADR cells. With SALL4 knockdown, ADMh accumulation rate of MCF-7/ADR cells was increased, while the expression of BCRP and c-myc was significantly decreased. Furthermore, silencing SALL4 also suppressed the growth of the xenograft tumors and reversed their resistance to ADMh in vivo.

**Conclusion:**

SALL4 knockdown inhibits the growth of the drug resistant breast cancer due to cell cycle arrest and reverses tumor chemo-resistance through down-regulating the membrane transporter, BCPR. Thus, SALL4 has potential as a novel target for the treatment of breast cancer.

## Background

Breast cancer, the most common neoplasm, is the leading cause of cancer-induced death in women worldwide [1, 2]. Surgery combined with adjuvant therapy, such as chemotherapy and radiotherapy, may have increased the patient survival rate in some way. Unfortunately, advanced and recurrent breast cancer still has very poor prognosis due to the primary or secondary resistance to chemotherapy drugs. Chemo-resistance that limits the therapeutic potential of anticancer drugs, resulting in failure of cancer treatment, still remains the main obstacle in terms of successful treatment of breast cancer [3, 4]. Therefore, novel effective attempts to overcome chemo-resistance are urgently needed in order to improve the outcome of patients with advanced and recurrent breast cancer.

The mechanism of chemo-resistance is very complex, but the overexpression of some ATP-binding cassette (ABC) transporters and cancer stem cells, are two main causes of chemo-resistance [5–8].Interestingly, recent studies have shown that sal-like 4 (SALL4) gene, a member of the mammal homologs of Drosophila homeotic gene spalt, is shared by stem cells with tumor cells promoting their proliferation and conferring drug resistance on them. SALL4 gene maps to chromosome 20q13 in human and encodes zinc-finger transcription factor maintaining self-renewal and pluripotency of embryonic stem cells through a variety of signaling pathways, transcription factors, and epigenetic modulators [9–16]. And SALL4 gene mutations are known to cause Okihiro syndrome, an autosomal dominant disease involving dysplasia of multiple organs [17]. In mice, SALL4 homozygous knockout is embryonic lethal, and Ma et al. [18] reported that SALL4 can function as an oncogene in leukemogenesis by using a transgenic mouse model with SALL4 overexpression in most organs. Thereafter, SALLL4 aberrant expression in hematopoietic stem cells, in the leukemic cells of AML patients, and in some solid tumors has been reported by various groups [19–22]. Up-regulating SALL4 in cancer cells can promote their proliferative and invasive abilities. The overexpression of SALL4 in tumor patients often correlates with adverse progression and poor prognosis. Besides, the expression of SALL4 is higher in drug resistant primary acute myeloid leukemic patients than those from drug-responsive cases. SALL4 characterizes a feature of drug resistance through the maintenance of side population cancer stem cells and affects the side population cells by regulation of ABC drug transport genes ABCG2 and ABCA3 in leukemic cells [23]. In liver cancer, SALL4 also enhances chemo-resistance by up-regulating ABCG2 [24]. In endometrial cancer, however, SALL4 promotes tumor chemo-resistance by activating c-myc to regulate MDR1/P-gp [25]. Furthermore, silencing SALL4 can enhance the chemo-sensitivity of tumor cells to anticancer drugs in liver cancer, endometrial cancer and lung cancer [26, 27]. Nevertheless, studies on the relation between SALL4 and drug resistance and chemotherapeutic sensitivity in breast cancer are still lacking.

The human breast cancer resistance protein (BCRP) is encoded by ABCG2 gene and it is a member of the G subfamily of the large ABC transporter superfamily [28, 29]. MDR1, also named ABCB1, encodes P-glycoprotein(P-gp) and it is one of the earliest ABC transporters to be identified. The high expression of MDR1 was found in the majority of breast cancer tissues and human breast cancer drug-resistant cells [30]. P-gp and BCRP were considered to be responsible for the majority of drug efflux in human cancer. A rise in their expression had something to do with a poor prognosis in many types of cancer. C-Myc gene encodes an evolutionarily conserved basic transcription factor and its overexpression is closely linked to chemotherapy resistance. In addition, overexpression c-myc can up-regulate the expression of BCRP in human mammary epithelial cells [31]. Therefore, we are interested in determining whether P-gp, BCRP and c-Myc are related with SALL4 involved in chemotherapy resistance in breast cancer.

In this study, we confirmed that SALL4 was overexpressed in the drug resistant cell line, MCF-7/ADR, and we down-regulated SALL4 gene in MCF-7/ADR through the lentiviral-mediated short hairpin RNA (shRNA) interference system. Then we examined the effects of SALL4 in MCF-7/ADR cells on proliferation and sensitivity to doxorubicin hydrochloride (ADMh) in vitro and in vivo, and explored the possible molecular mechanisms underlying these effects, to provide experimental evidence for the potential application of SALL4 gene as a therapeutic target in breast cancer.

## Methods

### Cell lines and culture

The human breast cancer cell lines, MCF-7, MDA-MB-231, SK-BR-3, ZR-75-1 and the human mammary epithelial cell line, HBL-100, were purchased from Cell Bank of the Chinese Academy of Science (Shanghai, China). Human multidrug resistant breast cancer cell line, MCF-7/ADR, was obtained from the Cancer Institute of Zhejiang University. HBL-100 was cultured in RPMI-1640 medium (Gibco-Life Technologies, Grand Island, NY, USA) supplemented with 10 % fetal bovine serum (FBS, Gibco) as well as 1 % penicillin–streptomycin (Gibco). The other cells were all cultured in Dulbecco modified Eagle medium (DMEM, Gibco), supplemented with 10 % FBS and 1 % penicillin–streptomycin. Cells were all passaged by trypsinization every 2–3 days and maintained at 37 °C in 5 % CO_2_. And all cells were cultured following the guidelines of Laboratory of Xinhua Hospital, affiliated to Shanghai JiaoTong University, School of Medicine, China.

### Drugs and antibodies

Doxorubicin hydrochloride (ADMh) and Verapamil were purchased from Sigma Chemical Co. (St. Louis, MO, USA). For in vitro studies, they were dissolved in Stroke-physiological saline solution (NS, NJCTT Pharmaceutical Co., Ltd, Nangjing, China) to create a stock solution (10 mmol/L), which was stored at −20 °C. To prepare working solutions, the stock solution was further diluted with culture media to yield the desired ADMh concentration. Primary antibodies against P-gp (goat-anti-rabbit) were purchased from Sigma Chemical Co. (St. Louis, MO, USA). Primary antibodies against c-myc, SALL4, BCRP, GAPDH and secondary antibodies (goat-anti-rabbit) were purchased from Cell Signaling Technology (Danvers, MA, USA).

### Lentivirus-mediated RNA interference and transfection

According to the gene data of SALL4 (NM‑020436) in GenBank, the designed sequence of the short hairpin RNA (shRNA) used to target SALL4 was 5′-GCCTTGAAACAAGCCAAGCTA-3′. And the sequence of the negative control shRNA was 5′-TTCTCCGAACGTGTCACGT-3′.The shRNAs were synthesized and inserted into the pGMLV-SC5 lentivirus core vector containing a cytomegalovirus-driven enhanced green fluorescent protein (GFP) reporter gene. Recombinant lentiviruses expressing SALL4-shRNA (Lv-shSALL4) or negative control shRNA (Lv-shNC) were produced by Genomeditech Co.Ltd (Shanghai, China). The experiment of MCF-7/ADR cells set up an experimental group (Lv-shSALL4), the negative control group (Lv-shNC) and blank control group (CON). MCF-7/ADR cells were infected with concentrated virus in serum-free medium. The supernatant was replaced with complete culture medium after 24 h. After being transfected for 96 h, mRNA and protein expression of SALL4 in the infected cells was validated by quantitative real-time PCR (qRT-PCR) analysis and western blot assays.

### Quantitative real-time PCR (qRT-PCR)

Total RNA was extracted from the control and transfected cells using Trizol reagent (Takara, Shiga, Japan) and reverse transcribed into cDNA with the M-MLV Reverse Transcriptase (Invitrogen, Carlsbad, CA). RNA expression was measured by qRT-PCR using the SYBR-Green method (Takara) according to the manufacturer’s instructions. The relative expression level of the target gene was calculated by 2^−ΔΔCT^ (ΔCT = CT^target^ − CT^GADPH^, ΔΔCT = ΔCT^target^ − ΔCT^con^) and normalized to the relative expression detected in the corresponding control cells, which was defined as 1.0 [32]. GAPDH was used as the referral gene. And HBL-100 cells was used as the control cells in calculating the relative expression of SALL4 among the six breast cell lines. Primer sequences were as follows: GAPDH forward, 5′-GAGAGACCCTCACTGCTG-3′; and reverse, 5′-GACTGGTAGATGACAAGGTGC-3′; SALL4 forward, 5′-CCAATAGTCAAGAAAGC-3′; and reverse, 5′-ATCGCTCCGACCTTCCATC-3′; ABCG2 forward, 5′-TTATCCGTGGTGTGTCTGGA-3′; and reverse, 5′-GATGATTGTTCGTCCCTGCT-3′; c-myc forward, 5′-GCTGCTTAGACGCTGGATTT-3′; and reverse, 5′-CGAGGTCATAGTTCCTGTTGG-3′; MDR1 forward, 5′-CCGCTGGTTTCCTTTAG-3′; and reverse, 5′-CTTCTTTGCTCCTCCATTGC-3′.

### Western blot analysis

Western blot analysis was carried out as described before [33]. Briefly, cellular proteins were extracted using lysis buffer (Beyotime, Shanghai, China) from the treated cells. Protein concentrations were quantified by the BCA protein assay reagent kit (Beyotime) according to the manufacturer’s instructions. Equal quantities of cellular proteins were resolved by SDS-PAGE and then electrophoretically transferred to nitrocellulose membranes (Millipore, Bedford, MA, USA). Each membrane was blocked with 5 % skim milk and immuneblotted with a primary antibody. After incubation with a secondary antibody, the bands were visualized by enhanced chemiluminescence (Millipore, Billerica, MA). GAPDH was used as the loading control.

### Cell viability assay

The cells were inoculated into 96-well plates and treated according to the experimental grouping requirements. The 10 ul of CCK-8 (Dojindo, Kumamoto, Japan) was added to each well of the plate, and the plates were maintained at 37 °C and 5 % CO_2_ for 3 h, and the absorbance at 450 nm (A450) was measured with a microplate reader (Bio-Tek, Winooski, VT, USA). The results were expressed as the average of three independent experiments. The absorbance values were used to draw growth curve and calculate the relative proliferation rate (RPR). The inhibition ratios (IR), resistance indices (RI) and the changes in IC50 values (the concentration of drug inhibiting 50 % of the cells) at 24 and 48 h were also calculated based on A450. RPR is the ratio of the A450 value of the tested day and that of the first day in a group cells. And RI is the ratio of IC50 values of resistant cells and that of parental cells. IR, PRP and RI were calculated according to the following formulas: PRP = A450 (the tested day)/A450 (the first day), IR = [1 − (A450treatment − A450blank)/(A450control − A450blank)] × 100 %, and RI = IC50(MCF-7/ADR cells)/IC50(MCF-7 cells).

### Cell cycle analysis

MCF-7/ADR cells infected by the recombinant lentiviruses or not were inoculated into 6 well plates for 48 h. Then, cells were harvested by trypsinization, washed twice in cold PBS, and fixed in 70 % ethanol at 4 °C overnight. Next, the cells were washed and resuspended in cold PBS and incubated in a solution of 10 mg/mL RNase and 1 mg/mL propidium iodide (Sigma-Aldrich) at 37 °C in the dark for 30 min. Finally, the samples were analyzed by flow cytometry (BD Biosciences). The percentage of cells in the G1, S, and G2/M phases was determined using Cell Quest acquisition software (BD Biosciences).

### Colony formation assay

Cells in the logarithmic growth phase were aliquoted as single cell suspension and 400 cells were placed into each well of 6-well plates (Corning, Corning, NY, USA). After adherence, cells were treated with ADMh (5 and 10 umol/L) for 12 h. Then, all plates were incubated for 14 days to allow colony formation. Next, cells were fixed with 4 % paraformaldehyde and stained with 0.1 % crystal violet (Sigma-Aldrich) for 20 min. After washing, the plates were air-dried, and stained colonies were photographed with a microscope (Leica, Wetzlar, Germany). The total number of colonies (>50 cells/colony) was counted.

### Analysis of intracellular accumulation of ADMh

The assay of intracellular accumulation of ADMh were conducted as previously described [34]. In short, the intracellular accumulation of ADMh in MCF-7/ADR cells and parental MCF-7 cells was measured by flow cytometry (BD Biosciences). The excitation and emission wavelengths of ADMh were 488 and 566 nm, respectively. The cells were incubated with ADMh (5 umol/L) for 2 h under normal cell culture conditions. Next, the cells were removed from the culture dishes by trypsinization and centrifuged and suspended in ice-cold PBS. Then, the samples were analyzed by flow cytometry (BD Biosciences). In another experiment, to show the effect of P-gp and BCRP inhibitor on the anthracycline drugs accumulation, cells were preincubated with Verapamil (5 umol/L) for 30 min and then incubated with ADMh (5 umol/L) for 2 h. Cellular drug fluorescence was measured using an argon laser at FL2 channel. For all samples 20,000 cells were counted and the analysis was performed using flow cytometry.

### Experimental animals

This study was approved by the ethics committee of Xinhua Hospital of Shanghai Jiaotong University,School of Medicine, China. Female BALB/c nude mice, 4–6 weeks old, were purchased from Shanghai SLAC Laboratory Animal Co., Ltd. (Shanghai, China). The mice were housed in groups of five in specific pathogen-free conditions following the guidelines of Laboratory Animals and the Ethics Committee of Xinhua Hospital, affiliated to Shanghai JiaoTong University. To explore the effects of SALL4 on tumor growth and drug resistance in vivo, MCF-7/ADR cells (3 × 10^6^) stably expressing Lv-shRNA suspended in 150 ul PBS were subcutaneously injected into the right axilla of the mice. On Day 7, these mice carrying xenograft tumor of MCF7/ADR cells infected with Lv-shSALL4 were randomly divided into two groups (5 mice/group). The first group received an intraperitoneally (i.p.) injection of NS every 3 days and the other group were administered an i.p. injection of ADMh (3 mg/kg) every 3 days. Simultaneously, these mice carrying xenograft tumor of MCF7/ADR cells infected with Lv-shNC were also randomly divided into two groups (5 mice/group) and subsequently treated as the Lv-shRNA group. On Day 20, all nude mice were anesthetized with CO_2_, and the tumor tissue was excised from mice and weighed.

### Statistical analysis

Statistical analyses were carried out using GraphPad Prism 5.0 software (La Jolla, CA, USA). All assays were performed independently three times and data were expressed as the mean ± SD. The independent Student’s *t* test was used to compare the means of two groups. The analysis of variance (ANOVA) test was performed in 2 × 2 factorial design to test a synergistic effect of shRNA-driven knockdown of SALL4 and drug treatment on tumor growth. The difference was considered statistically significant when *P* < 0.05.

## Results and discussion

### SALL4 is overexpressed in chemo-resistant breast cancer cell line MCF-7/ADR

**Fig. 1:**
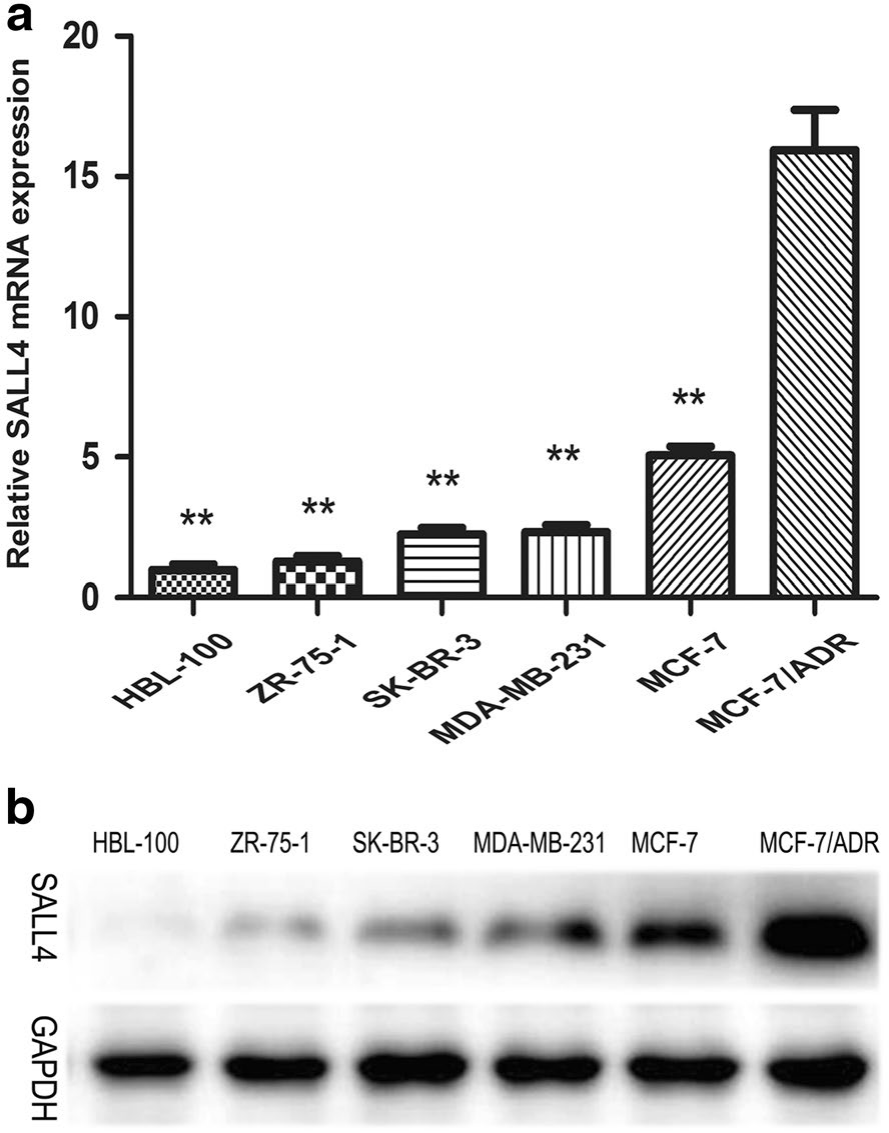
Expression of the transcription factor SALL4 (sal-like 4) in breast cell lines. **a** MRNA levels of SALL4 expressed in the indicated cell lines were evaluated by quantitative real-time PCR (qRT-PCR). Data are expressed as mean ± standard deviation (SD) of at least three independent experiments. **P < 0.01, when compared to MCF-7/ADR cells; and **b** protein levels of SALL4 were evaluated by western blot in the indicated cell lines

To assess the role of SALL4 in the drug resistant breast cancer cells, we detected the endogenous expression of SALL4 in the normal mammary epithelial cell line HBL-100 and five breast cancer cell lines including MCF-7, MDA-MB-231, SK-BR-3, ZR-75-1 and MCF-7/ADR by qRT-PCR and Western blot. MCF-7, MDA-MB-231, SK-BR-3 and ZR-75-1 cell lines are sensitive to chemotherapy drugs such as anthracycline, taxane and so on. But MCF-7/ADR cells are resistant to many drugs, despite the diversity in their chemical structures and mechanisms of action. And it was established from MCF-7cell line by exposing to adriamycin with stepwise increasing concentration [35]. The relative expression level of SALL4 was significantly higher in MCF-7/ADR cells compared with that in the other five cell lines (*P* < 0.05, Fig. 1a). And the results of western blot of SALL4 were consistent with the results of mRNA (Fig. 1b). Previously, gain- and loss-of-function studies have revealed that overexpression of SALL4 was correlated with chemo-resistance in myeloid leukemia, endometrial cancer, lung cancer and liver cancer. Taken together, these results illustrate that SALL4 may also play an important role in regulating the resistance to chemotherapeutics in breast cancer.

### Knockdown of SALL4 inhibits cell proliferation

**Fig. 2:**
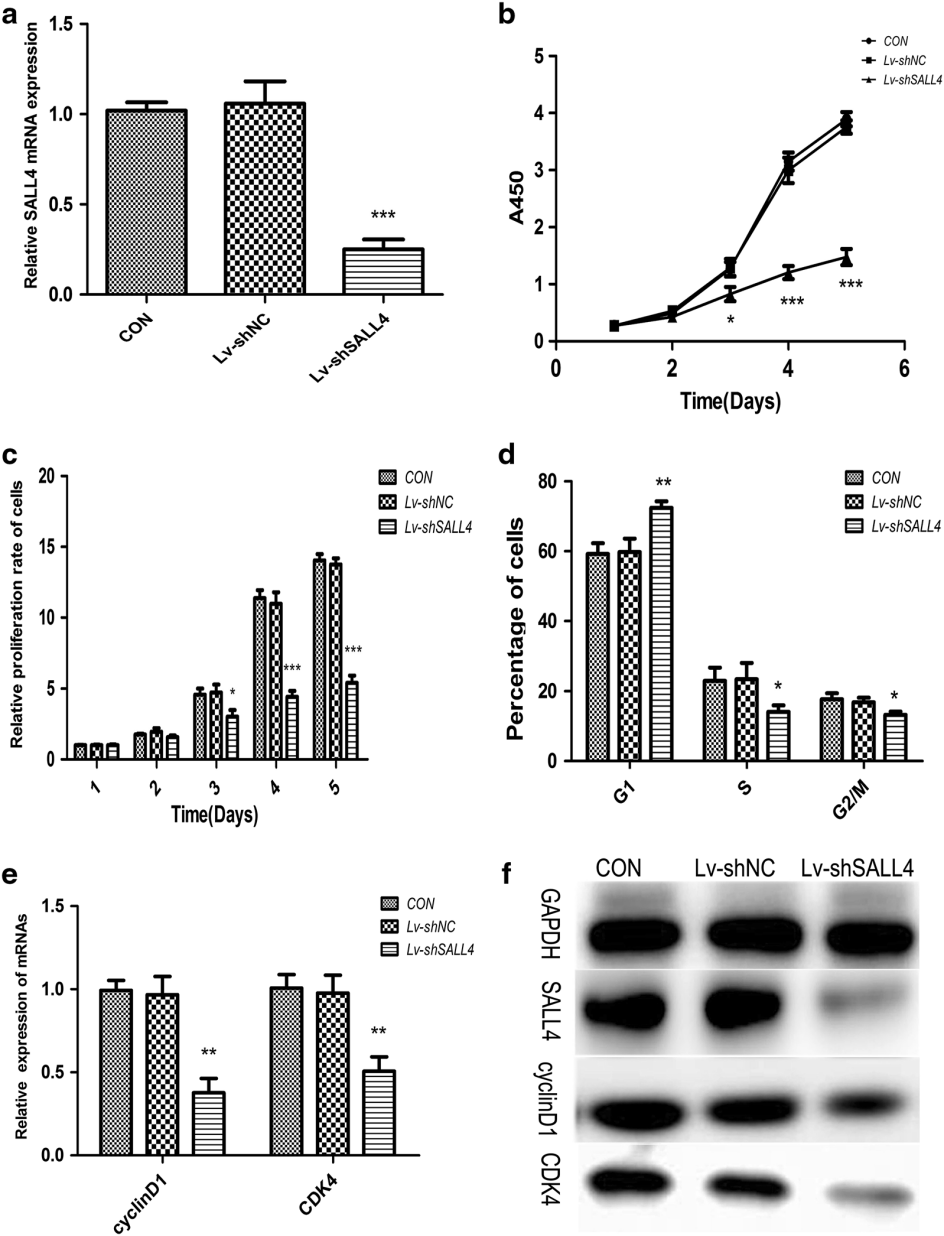
Down-regulation of SALL4 inhibits proliferation and changes cell cycle distributions in MCF-7/ADR cells. **a** MRNA levels of SALL4 in the indicated cells were assessed by qRT-PCR (***P < 0.001); and **b** growth curves of MCF-7/ADR cells and **c** the relative proliferation rate of the cells with or without SALL4 knockdown (*P < 0.05 and ***P < 0.001); and **d** cell cycle distribution in percentages of different groups (*P < 0.05 and **P < 0.01); and **e** effects of SALL4 on the mRNA levels of cyclinD1 and CDK4 genes. GAPDH was used as the referral gene. (**P < 0.01); and **f** the levels of indicated proteins, GAPDH was used as the loading control, and the experiments were performed in triplicate

To explore the effects of SALL4 on the chemo-resistant breast cancer, we established a lentiviral system expressing shRNA to transfect MCF-7/ADR cells. The transfection efficiency was confirmed by qRT-PCR (Fig. 2a) and western blot (Fig. 2f).SALL4 mRNA detection in the cells showed the shRNA sequence targeting SALL4 significantly inhibited SALL4 expression compared with the CON group (*P* < 0.001). On the contrary, the negative control sequence (Lv-shNC) did not show statistically effect on the target gene (*P* > 0.05). The results of western blot of SALL4 also coincided exactly with the results of mRNA. These data suggest that we have successfully down-regulated SALL4 in MCF-7/ADR cells by the approach lentivirus-mediated shRNA interference.

By comparing the growth curves of MCF-7/ADR cells with or without SALL4 knockdown, SALL4 knockdown seemed to significantly inhibit the cell viability. The cell viability in SALL4 knockdown group was significantly lower than that in the CON group at the third day (*P* < 0.05), and the inhibitory effect on cell viability became more obvious at the fourth and fifth day (*P* < 0.001, Fig. 2b). The relative proliferation rate also indicated cell proliferation was markedly reduced in Lv-shSALL4 group since the third day when compared to the CON group (*P* < 0.05, Fig. 2c).But there was no observable difference between the CON group and the Lv-shNC group (*P* > 0.05). According to those data, SALL4 knockdown may exert a significant influence on MCF-7/ADR cell viability and proliferation.

Arresting cell cycle progression can inhibit the proliferation of cells [36]. To investigate the potential molecular mechanisms of SALL4 on the cell proliferation, we examined the cell-cycle analyses in MCF-7/ADR using flow cytometry, and the results showed that knockdown of SALL4 significantly changed cell cycle distributions in MCF-7/ADR cells by increasing the percentage of cells in the G1 phase accompanied with reducing the percentage of cells in the S and G2/M phase (*P* < 0.05, Fig. 2d), demonstrating typical cell cycle arresting at the G1 phase. In terms of all of the detected indices, there were no difference between the Lv-shNC group and the CON group (*P* > 0.05). These findings are consistent with the previous reports that down-regulation of SALL4 suppresses the growth of cancer cells and induces G1 phase arrest [37, 38]. We also evaluated the levels of cycle-related proteins cyclinD1 and cyclin-dependent kinase 4 (CDK4) by qRT-PCR and western blot analysis. CyclinD1 is one of the main factors in cell cycle regulation [39, 40], which binding with cyclin-dependent kinase, then leading G1 phase cells into S phase and promoting cell proliferation. A significant decrease of mRNAs and proteins (cyclinD1 and CDK4) was observed in MCF-7/ADR cells in the target gene knockdown group (Fig. 2e, f), indicating that knockdown SALL4 induced G1 phase arrest in these cells. These results suggest that SALL4 has an impact on the proliferative activity of the resistant breast cancer cells through cell cycle arrest.

### Silencing SALL4 re-sensitizes MCF-7/ADR cells to ADMh in vitro

**Fig. 3:**
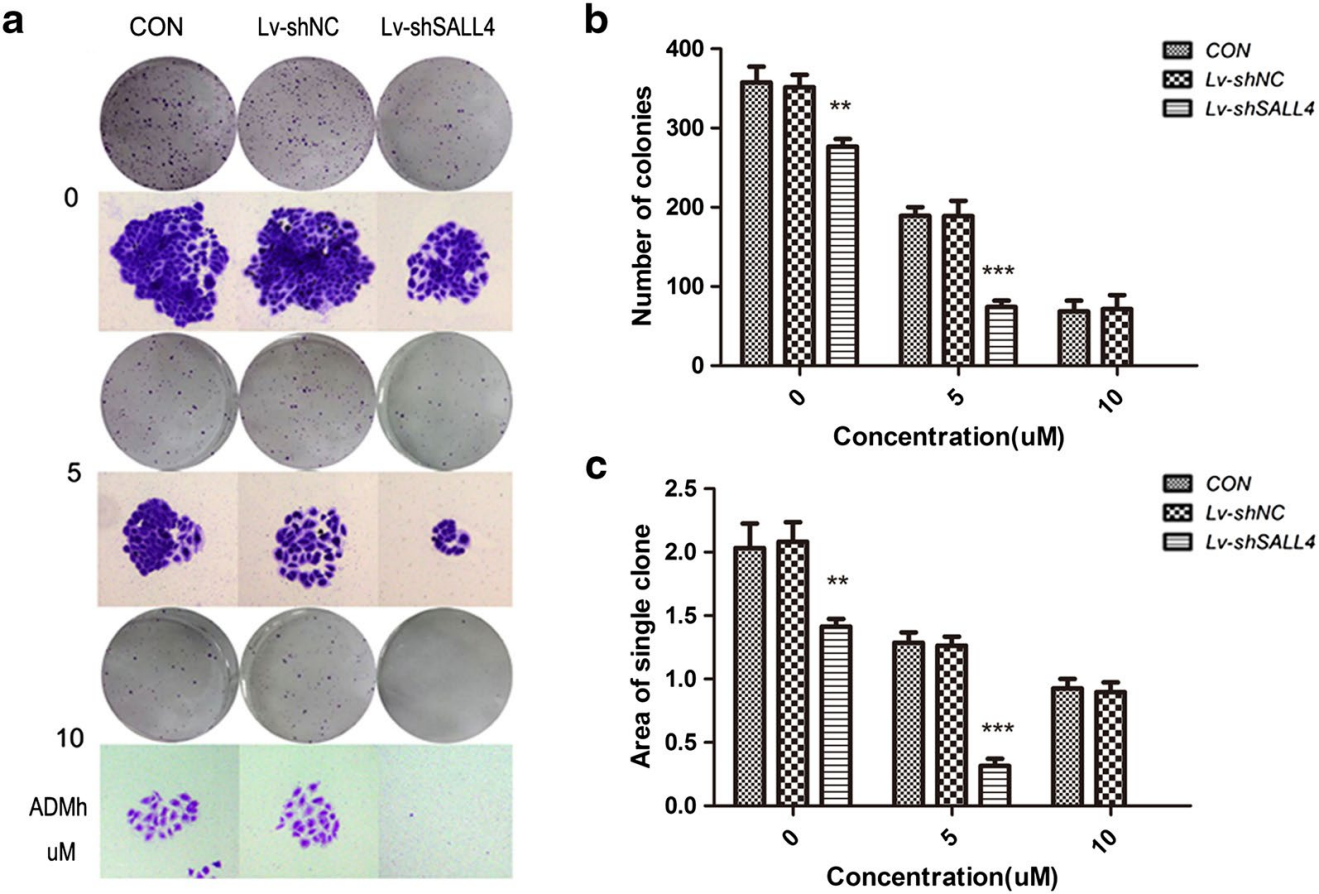
Synergistic cytotoxic effect of SALL4 knockdown and ADMh on MCF-7/ADR cells. **a** The photo-micrographic differences, and **b**, **c** influence of colonies (mean ± SD, n = 3) on colony formation are shown. All data were from three independent experiments. MCF-7/ADR cells were treated with various concentration of ADMh (0, 5 and 10 μmol/L) for 12 h and were allowed to form colonies in fresh medium for 14 days, **P < 0.01, ***P < 0.001, when compared to the CON group

Silencing SALL4 in MCF-7/ADR cells evidently enhanced its sensitivity to ADMh. As shown in the Table 1, the IC50 values of ADMh at 24 and 48 h in the Lv-shSALL4 group decreased from (152.57 ± 2.33) and (29.66 ± 1.14) umol/L to (42.09 ± 3.42) and (6.48 ± 0.42) umol/L, respectively. And RI also became apparently lower than that in the CON group (*P* < 0.01). Besides this, a marked inhibition of colony formation ability was observed in the colony formation assay. The colony count and area were smaller in Lv-shSALL4 group (*P* < 0.01, Fig. 3). And the ANOVA test indicated there was a synergistic effect of SALL4 knockdown and ADMh treatment on inhibition of colony formation ability (*P* < 0.05). The results mentioned above suggest that knockdown SALL4 can re-sensitize MCF-7/ADR cells to ADMh and reduce it growth.

**Table 1:** The analysis of the IC50 and RI of MCF-7 and MCF-7/ADR cells towards ADMh

Intervention time/h	IC50 (uM)				Resistance index (RI)		
	MCF-7	MCF-7/ADR			MCF-7/ADR		
		CON	Lv-shNC	Lv-shSALL4	CON	Lv-shNC	Lv-shSALL4
24	1.06 ± 0.09	152.57 ± 2.33	149.03 ± 3.28	42.09 ± 3.42**	143.93 ± 2.20	140.60 ± 3.60	39.70 ± 33.23**
48	0.22 ± 0.01	29.66 ± 1.14	30.01 ± 1.88	6.48 ± 0.42**	134.82 ± 5.17	136.41 ± 5.40	29.63 ± 1.59**

Lv-shSALL4 group vs. CON group

** *P* < 0.01

**Fig. 4:**
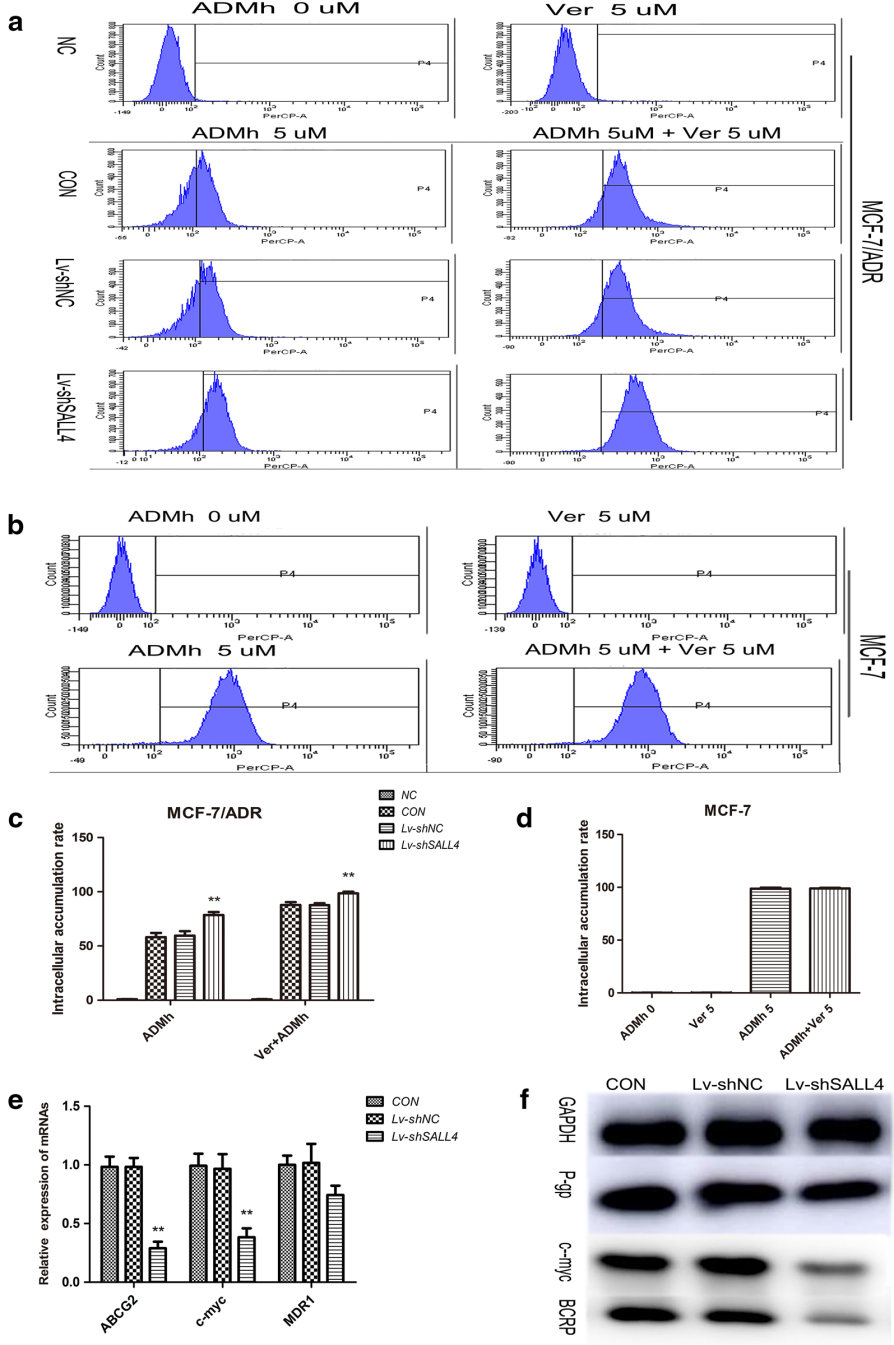
ADMh accumulation rates of MCF-7/ADR and MCF-7 cells and effects of SALL4 on ABCG2, MDR1 and c-myc. **a**, **b** The intracellular accumulation of ADMh in the tested cells was analyzed by flow cytometry, and **c**, **d** the intracellular accumulation of ADMh in percentages of different groups. All data were from three independent experiments. **P < 0.01, when compared to the CON group; and **e** effects of SALL4 on the mRNA levels of indicated genes. GAPDH was used as the referral gene. All data were from 3 independent experiments. **P < 0.01, when compared with the CON group; and **f** effects of SALL4 on the levels of indicated proteins. Results were representative of 3 independent experiments. GAPDH was used as the loading control

The cytotoxic effect of anticancer drugs is associated with the dose which can be accumulated in the cells [41]. In order to investigate the potential mechanisms of SALL4 and drug resistance of MCF-7/ADR cells, we detected intracellular accumulation rate of ADMh in MCF-7/ADR and parental MCF-7 cells. As shown in Fig. 4a, c, a markedly higher intracellular accumulation of ADMh was observed in the cells of Lv-shSALL4 group compared with the CON group (*P* < 0.01). Moreover, we also found that the intracellular accumulation of ADMh in the Verapamil pretreated cells was higher in MCF-7/ADR cells. But the ADMh accumulation rate was similar in parental MCF-7 cells with Verapamil pretreated or not (*P* > 0.05, Fig. 4b, d). Furthermore, the ANOVA test indicated that knockdown of SALL4 had synergy with Verapamil to increase ADMh accumulation rate in MCF-7/ADR cells (*P* < 0.05).We also investigated the effect of SALL4 on membrane transporters, BCRP and P-gp, as well as c-myc by qRT-PCR and western blot. With SALL4 knockdown, the expression of both c-myc and ABCG2/BCRP in MCF-7/ADR cells decreased significantly (*P* < 0.01). But with SALL4 knockdown, the expression of MDR1/P-gp only had a slight declining tendency, when compared to the CON group (*P* > 0.05, Fig. 4e, f). In other words, the function of ABCG2 and c-myc in SALL4-knocked-down MCF-7/ADR cells was effectively inhibited, while the function of MDR1in SALL4-knocked-down MCF-7/ADR cells was not significantly inhibited. Verapamil is a competitive inhibitor of both P-gp and BCRP, and they are ABC trans-membrane efflux proteins sharing the ability to actively extrude drugs and toxins out of the cells, which may conduce to increasing the drug resistance in cancer cells. ADMh is the substrate of both P-gp and BCRP. The intracellular accumulation rate of ADMh will increase when anyone of the trans-membrane proteins is inhibited. In view of this, we assumed that the accumulation rate of SALL4-knocked-down MCF-7/ADR cells was higher than the cells without SALL4 knockdown due to ABCG2/BCRP down-regulation. And the accumulation rate of SALL4-knocked-down MCF-7/ADR cells was increased again by Verapamil treatment caused by MDR1/P-gp inhibition. This was consistent with Yhee et al. [42], who reported drug accumulation rate increased in MCF-7/ADR cells by P-gp inhibition. However, the results of MCF-7 cells were not in agreement with Rogalska et al. [43], who reported that doxorubicin accumulation rate in MCF-7 cells preincubated with Verapamil increased higher. The reason for this difference probably was that the concentration, 5-umol/L of ADMh, was a supersaturated concentration for MCF-7 and the membrane transport proteins cannot continue extruding ADMh out of cells in our experiment. According to these data, we speculated SALL4 conferred breast cancer cells on drug resistance, at least partly through manipulating ABCG2 and c-myc genes. Knockdown SALL4 of MCF-7/ADR cells can enhance intracellular ADMh accumulation, leading to a higher therapeutic effect of ADMh.

### SALL4 down-regulation re-sensitizes drug-resistant tumor to chemotherapy in vivo

**Fig. 5:**
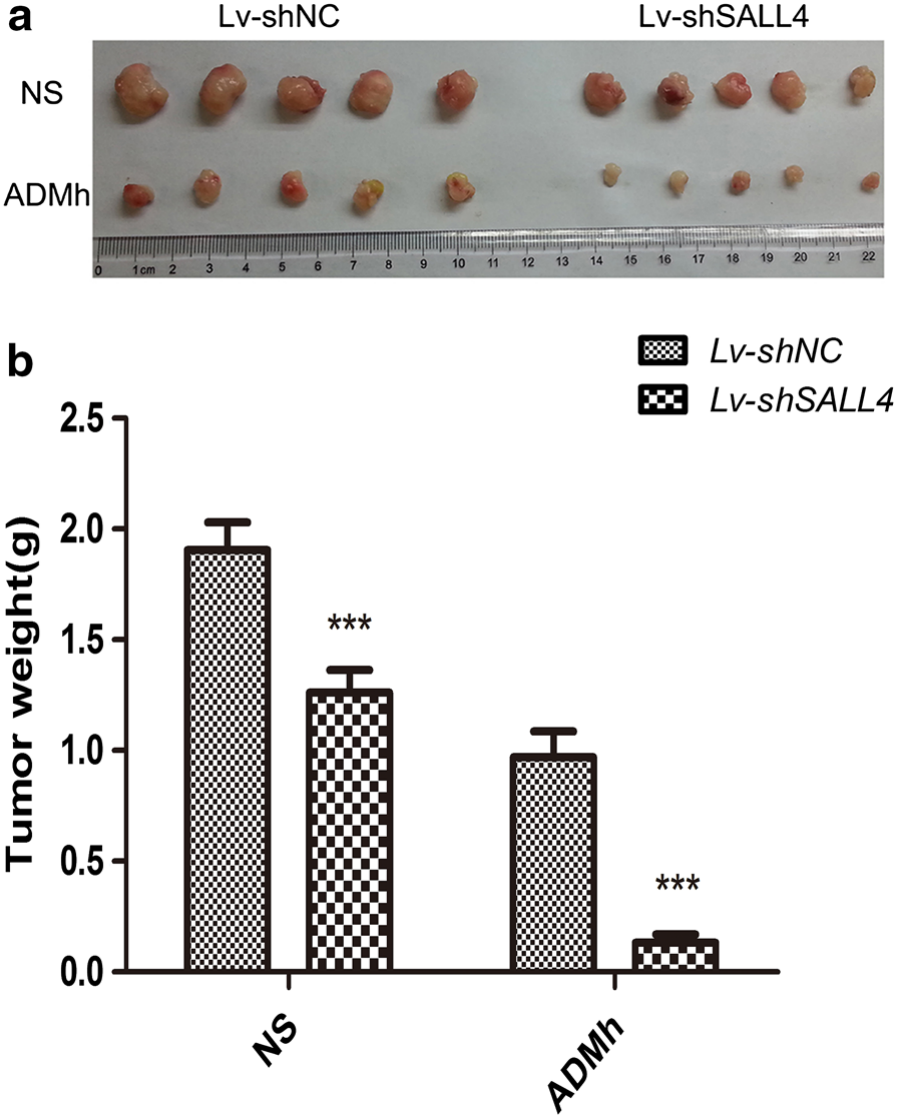
Knockdown of SALL4 re-sensitizes the in vivo tumor to chemotherapy. **a** Tumors were excised from the animals and photos of them were presented to show the sizes of the resulting tumors; and **b** tumors were weighed. ***P < 0.001, when compared to the Lv-shNC group

To investigate the effects of SALL4 down-regulation on tumor growth and drug resistance in vivo, stroke-physiological saline solution (NS) or ADMh (3 mg/kg) were intraperitoneally injected into nude mice carrying subcutaneous MCF-7/ADR tumor every 3 days for up to 20 days. The tumors removed from these animals were shown in Fig. 5a, and their mean weights were provided in Fig. 5b. The results showed an obvious inhibition of tumor growth in mice with SALL4 knockdown compared with the Lv-shNC group (*P* < 0.001). And the ANOVA test indicated SALL4 knockdown had potent synergy with ADMh to suppress the tumor growth in vivo (*P* < 0.05). Those were consistent well with the results of colony formation assay in vitro that knockdown of SALL4 can reduce the chemo-resistance and proliferation of MCF-7/ADR cells.

In order to increase the efficacy of breast cancer therapy, it is of great importance to identify candidate genes that are essential in cancer cell proliferation and drug resistance. SALL4, a stem cell factor plays vital roles in development and tumorigenesis. Its expression has been previously linked with tumor treatment status and drug resistance. SALL4 knockdown affects tumor growth in various malignancies including breast cancer. However, whether up-regulation of SALL4 is related with chemo-resistance of breast cancer and the underling mechanisms remained unclear. In this study, using multidrug resistant breast cancer cells MCF-7/ADR and its parental MCF-7 cells, we demonstrated that SALL4 was overexpressed in MCF-7/ADR cells. Knockdown of SALL4 in MCF-7/ADR decreased its proliferation and induced G1 phase arrest accompanied by down-regulation of cell cycle related proteins (cyclinD1 and CDK4). Analyses of the IC50 values and resistance indices indicated knockdown of SALL4 enhanced the chemo-sensitivity of MCF-7/ADR cells to ADMh. The observed difference in cytotoxicity and intracellular accumulation of ADMh might be related to the inhibition of SALL4 affecting the function of BCRP, c-myc and P-gp. The strongest suppression of tumor growth in vivo observed in SALL4 knockdown combined with ADMh group suggested knockdown of SALL4 and ADMh had synergistic inhibition of tumor growth. In summary, our study provided solid evidence that knockdown of SALL4 can significantly inhibit the proliferative captivity and reverse the drug resistance of breast cancer in vitro and in vivo; and these results suggest that SALL4 interference may have a profound effect on the chemotherapy of breast cancer.

## Conclusions

In conclusion, this study suggests that knockdown of SALL4 inhibits the proliferation of MCF-7/ADR cells through arresting the cell cycle in the G1 phase and that down-regulation of SALL4 reverses the drug resistance of breast cancer by reducing the expression of ABCG2 and c-myc. However, further mechanisms of SALL4 affecting tumor growth and drug sensitivity of tumor to chemotherapy are required to investigate.

## Abbreviations

SALL4: sal-like 4
shRNA: short hairpin RNA
ADMh: doxorubicin hydrochloride
NS: stroke-physiological saline solution
FBS: fetal bovine serum
Ver: verapamil
P-gp: P-glycoprotein
BCRP: breast cancer resistance protein
Lv-shSALL4: the experimental group
Lv-shNC: the negative control group
CON: the blank control group
h: hour
RPR: the relative proliferation rate
IR: the inhibition ratios
RI: resistance index
IC50: the concentration of drug inhibiting 50 % of the cells
